# The Integrin Receptor in Biologically Relevant Bilayers: Insights from Molecular Dynamics Simulations

**DOI:** 10.1007/s00232-016-9908-z

**Published:** 2016-07-27

**Authors:** Antreas C. Kalli, Tomasz Rog, Ilpo Vattulainen, Iain D. Campbell, Mark S. P. Sansom

**Affiliations:** 1 Department of Biochemistry, University of Oxford, South Parks Road, Oxford, OX1 3QU UK; 2 Department of Physics, Tampere University of Technology, P.O. Box 692, 33101 Tampere, Finland; 3 MEMPHYS – Center for Biomembrane Physics, University of Southern Denmark, 5230 Odense M, Denmark

**Keywords:** Integrin, Talin, Molecular dynamics simulations, Lipid diffusion

## Abstract

Integrins are heterodimeric (αβ) cell surface receptors that are potential therapeutic targets for a number of diseases. Despite the existence of structural data for all parts of integrins, the structure of the complete integrin receptor is still not available. We have used available structural data to construct a model of the complete integrin receptor in complex with talin F2–F3 domain. It has been shown that the interactions of integrins with their lipid environment are crucial for their function but details of the integrin/lipid interactions remain elusive. In this study an integrin/talin complex was inserted in biologically relevant bilayers that resemble the cell plasma membrane containing zwitterionic and charged phospholipids, cholesterol and sphingolipids to study the dynamics of the integrin receptor and its effect on bilayer structure and dynamics. The results of this study demonstrate the dynamic nature of the integrin receptor and suggest that the presence of the integrin receptor alters the lipid organization between the two leaflets of the bilayer. In particular, our results suggest elevated density of cholesterol and of phosphatidylserine lipids around the integrin/talin complex and a slowing down of lipids in an annulus of ~30 Å around the protein due to interactions between the lipids and the integrin/talin F2–F3 complex. This may in part regulate the interactions of integrins with other related proteins or integrin clustering thus facilitating signal transduction across cell membranes.

**Electronic supplementary material:**

The online version of this article (doi:10.1007/s00232-016-9908-z) contains supplementary material, which is available to authorized users.

## Introduction

The function of cell membrane requires the interplay of a lipid bilayer and the proteins embedded within it. Developments in lipidomics (Wenk 2010) and in membrane biophysics and biochemistry (Landreh and Robinson 2015; Quinn 2012; Spillane et al. 2014) have revealed that the diversity of lipids plays a key role in the physical and biological properties of cell membranes. In particular, a number of studies have demonstrated that variations in lipid types in cell membranes are important in cellular functions including transmembrane (TM) signalling (Coskun et al. 2011; Kawashima et al. 2009; Michailidis et al. 2011) and membrane protein trafficking (Bretscher and Munro 1993).

Protein/lipid interactions and the resultant dynamic organization of membranes occur on scales of nanometres and microseconds. These scales can be investigated using molecular dynamics (MD) simulations (Khalili-Araghi et al. 2009; Lindahl and Sansom 2008; Stansfeld and Sansom 2011). All-atom MD simulations have provided insights into protein/lipid interactions, although they remain computationally expensive, reflecting the relatively slow diffusion rates of lipids (typically ~10^−12^ m^2^/s (Gaede and Gawrisch 2003)) in membranes (Kaiser et al. 2011; Lingwood et al. 2011; Niemela et al. 2007). Consequently, lower resolution techniques such as coarse-grained (CG) simulations have been extensively used to study lipid dynamics and organization both in bilayers without a protein component (Ingólfsson et al. 2014; Koldsø et al. 2014) and in the presence of integral membrane proteins (Koldsø and Sansom 2015; Schafer et al. 2011). MD simulations have provided valuable insights into the dynamics and lipid interactions of a number of classes of TM receptors, including GPCRs (Johnston and Filizola 2011) and receptor tyrosine kinases, the latter exemplified by the EGFR receptor (Abd Halim et al. 2015; Arkhipov et al. 2013; Kaszuba et al. 2015). In particular, MD simulations are starting to reveal how a number of TM receptors may operate within a local nano-environment, which differs in its lipid composition from that of the membrane as a whole as a consequence of preferential interactions with certain lipid species in the membrane (Gambin et al. 2006). Given the diversity of different types of receptors present within mammalian cell membranes (Gambin et al. 2006), it is therefore important to understand their interactions with the lipids around them.

**Fig. 1:**
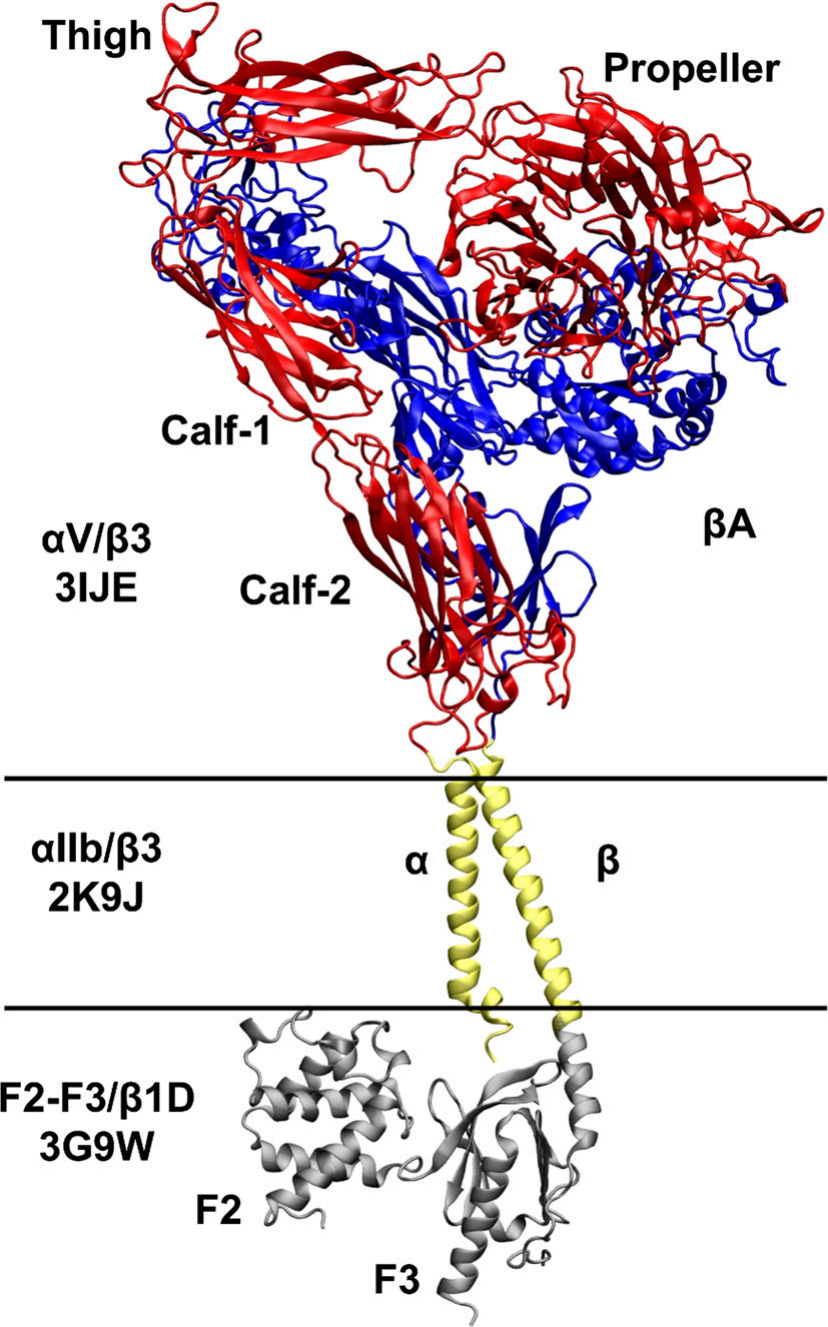
Model of the integrin/F2–F3 complex. The β subunit comprised β3 G1-P685 residues/β3 685-718 residues/β1D 754-787 residues whereas the α subunit comprised αV F1-P963 residues/αIIb P965-P998 residues. The part of the structure corresponding to the αIIbβ3 structure (PBD:2K9J) is shown in* yellow*, the F2–F3/β1D (PDB:3G9W) is in *grey* and the integrin ectodomain (PDB:3IJE) is in orange (Color figure online)

Integrins are heterodimeric (αβ) receptors which are involved in cell surface adhesion and in a number of signalling pathways (Anthis and Campbell 2011). They are crucial to many cellular processes, and are potential therapeutic targets for a number of diseases such as thrombosis, inflammation and cancer (Desgrosellier and Cheresh 2010). In mammals there are 24 different integrins. Each consists of a large ectodomain (Xiong et al. 2009), a dimeric TM domain (Lau et al. 2009; Yang et al. 2009) and a largely unstructured cytoplasmic domain (Anthis et al. 2009) (Fig. 1). The inactive state of integrins is maintained mainly via interactions in their TM region. When in the inactive state, the integrin ectodomain adopts a bent conformation, such that there is a “knee” in the α subunit between the Calf-1 and Thigh domains, and in the β subunit between the EGF1 and Hybrid domains (Fig. 1). Binding of the head domain of the cytosolic protein talin to the cytoplasmic regions of the β integrin (Anthis et al. 2009; Calderwood 2004; Kalli et al. 2013a) has been shown to result in reorientation of the integrin TM helices (Kalli et al. 2011a). This in turn may result to substantial conformational changes in the integrin ectodomain thus resulting in integrin inside-out activation (Shattil et al. 2010). The head domain of talin consists of four subdomains (F0 to F3) (Elliott et al. 2010) which are connected with flexible linkers. The crystal structure of the talin head domain suggests that the four subdomains are in a linear arrangement. Computational studies, however, have shown that when the talin head domain associates with the membrane it adopts a V-shaped configuration due to the rotation of the F0–F1 pair relative to the F2–F3 pair (Kalli et al. 2013a). The F2 and F3 subdomains have positively charged patches (or loops) which interact with the lipids of the intracellular leaflet of the cell membrane (Anthis et al. 2009; Elliott et al. 2010), and these interactions are suggested to be important for integrin activation (Anthis et al. 2009; Elliott et al. 2010; Kalli et al. 2010, 2011a).

The structural data for integrins (Anthis et al. 2009; Lau et al. 2009; Xiong et al. 2009) allow the construction of models of the complete integrin receptor within a lipid bilayer (Fig. 1). The mammalian plasma membrane has an asymmetric distribution of lipids with sphingolipids (mainly sphingomyelin) and sterols (i.e. cholesterol) but not glycerolipids in high concentrations in the outer leaflet (van Meer et al. 2008). Note, however, that sterols can also be found in the inner leaflet of the plasma membrane (Mondal et al. 2009). Sphingomyelin has a polar headgroup with saturated lipid tails and a trans double bond between the C4 and C5 atoms in the acyl chain (Barenholz and Thompson 1999; Ramstedt and Slotte 2002) and may be directly involved in TM signalling (Hannun and Obeid 2008). Cholesterol increases the thickness and stiffness of lipid bilayers (Lundbaek et al. 2003) by increasing the packing density of lipids (Falck et al. 2004; Hofsaß et al. 2003; Ohvo-Rekilä et al. 2002; Róg and Vattulainen 2014) Furthermore, it may facilitate receptor activity (Lingwood et al. 2011), bring about sorting of TM helices (Kaiser et al. 2011), and influence the lateral pressure of a membrane (Samuli Ollila et al. 2007).

Computational studies of the integrin receptor to date have focused on studying the dynamics of the integrin receptor using either fragments of the integrin/talin complex and/or the receptor embedded in simple, usually single lipid species, bilayers. In particular, several computational studies have examined the isolated talin head domain (or subdomain fragments of the head domain) in bilayers with zwitterionic PC and/or negatively charged PS or PC/PG lipid model membranes (Arcario and Tajkhorshid 2014; Kalli et al. 2010, 2013a). The talin head domain/integrin TM (αβ) domain complex was also studied using microsecond-scale atomistic and coarse-grained molecular dynamics simulations probing the effect of the talin head on the packing and the interactions of the integrin TM and cytoplasmic regions (Kalli et al. 2011a, 2013a; Provasi et al. 2014). The TM domains of different classes of integrin e.g. αIIbβ3 (Kalli et al. 2011b) and αLβ2 (Chng and Tan 2011; Vararattanavech et al. 2010) have also been studied using simulations, revealing the crucial role of the Gx_3_G motif and of the interactions in the integrin TM outer membrane clasp in the packing of the integrin TM region. A number of molecular dynamics studies (e.g. using steered molecular dynamics simulations) based on structures of the isolated integrin ectodomain (with no lipid bilayer present) have investigated conformational changes required for the transition of the ectodomain from a folded inactive to a more extended active state (Jallu et al. 2014; Murcia et al. 2008; Puklin-Faucher et al. 2006; Zhu et al. 2008). Recently, steered molecular dynamics simulations suggested that β integrin TM domain homo-oligomerization may regulate integrin clustering (Mehrbod and Mofrad 2013). Short all-atom simulations of αIIbβ3 integrin in a PC bilayer have been used to explore its interaction with RGD peptides and the effect of talin (Mehrbod et al. 2013).

These simulation studies have been performed either in simple symmetric lipid bilayers, or without a bilayer present. They thus represent a considerable simplification of the environment in which this receptor functions in vivo. It has been shown that the lipid environment plays a pivotal role in talin-mediated integrin activation (Elliott et al. 2010; Ye et al. 2010). It has been also suggested that negatively charged annular lipids stabilize the integrin αIIbβ3 TM domain (Schmidt et al. 2015). The presence of anionic lipids and or/cholesterol may influence the binding of extracellular proteins to integrin ectodomains (Conforti et al. 1990; Smyth et al. 1992). Therefore, simulation of the interactions and dynamics of an integrin receptor in a more complex lipid bilayer will provide a significantly closer approximation to the behaviour of the protein in its in vivo environment.

In the current study our goal is two-fold: (i) we wish to significantly extend the complexity of integrin simulations by studying a complete receptor in complex with its activating protein (talin) in lipid bilayers whose lipid composition mimics aspects of a mammalian plasma membrane and (ii) to examine how the presence of a signalling receptor changes the structure and dynamics of its local lipid environment. Extended (10 μs) CG-MD simulations enable us to examine the dynamics and interactions of the integrin/talin complex in different lipid bilayers containing cholesterol, phospholipids and sphingomyelin. The results of these simulations are analysed in terms of the effects of the integrin/talin complex on the local organization and dynamics of lipids in the membrane. The results demonstrate that the integrin/talin complex influences the dynamic organization of lipids in both the outer and inner leaflets of the bilayer, and that the diffusion of lipids in the vicinity of the protein is slowed down.

## Materials and Methods

### Integrin/Talin F2–F3 Complex Modelling

The complete integrin receptor was modelled using the integrin TM αIIb/β3 NMR structure (PDB:2K9J) (Lau et al. 2009) plus the αVβ3 ectodomain structure (PDB:3IJE) (Xiong et al. 2009). During the modelling, the αΙΙb TM region was oriented at an angle of ~30° relative to the axis of the α subunit ectodomain leg (i.e. Calf-1 and Calf-2 domains) to satisfy available experimental data (Fig. 1) (Nogales et al. 2009; Xiong et al. 2009). Modelling was done using Modeller (Fiser and Sali 2003) and the relative orientation of the two structures during the modelling was restrained. To construct the integrin α subunit, the two structures were connected at residues αIIbP965 (PDB:2K9J) and αVP963 (PDB:3IJE). To construct the integrin β subunit, the structures were connected at residues β3P685 (PDB:2K9J) and β3P685 (PDB:3IJE). This resulted in a structure with αVF1-P963/αIIbP965-P998 and β3G1-P685/β3P685-F727 subunits (Fig. 1). We note that the αVQ839 to αVG867 region that is missing from the crystal structure is also missing from our model. To model the conformation of the region between the TM region and the ectodomain, for which there are no available structural data (residues: α956–965 and β685–694 in Fig. 1), a structure prediction was performed using PSIPRED (Buchan et al. 2010), which suggested that both regions are unstructured (Xiong et al. 2009). The resulting model structure was energy minimized to remove any steric clashes. The integrin/talin complex was constructed by aligning overlapped residues at the TM region of the integrin receptor model (2K9J part of the structure; Fig. 1) with the TM region of the F2–F3/β1D (PDB:3G9 W) (Anthis et al. 2009).

### Lipid Bilayers

Bilayers (see Table 1) were prepared by performing self-assembly coarse-grained MD simulation (SA-CGMD) (Scott et al. 2008). In these simulations the lipids were randomly placed in a simulation box and solvated with coarse-grained water molecules and ions to neutralize the system. Subsequently, a production simulation was performed for 200 ns. After the first 10 to 15 ns of simulation the bilayer was formed. Initially, in the SA-CGMD simulations, only a small portion of the final lipid bilayer was prepared and after the end of the simulation, this portion was replicated in the x- and y-axis to reach the final bilayer size. A further 200 ns of simulation was performed to remove any discontinuities in the newly formed larger bilayer. In order to construct an asymmetric lipid bilayer, two bilayers were constructed, one with the lipid composition of the inner and one with the composition of the outer leaflet of an asymmetric bilayer (*Symm_I* and *Symm_O* bilayers in Table 1). To generate an initial model of an asymmetric bilayer a leaflet from the *Symm_O* bilayer and a leaflet from the *Symm_I* bilayer were combined. After the insertion of the protein, the *Symm_O* contained 452 POPC lipids, 459 SM lipids and 474 cholesterol molecules and the *Symm_I* bilayer contained 379 POPC lipids, 248 POPS lipids, 313 POPE lipids and 254 cholesterol molecules. The asymmetric bilayer contained 193 POPC lipids, 261 SM lipids and 220 cholesterol molecules in the outer leaflet and 240 POPC lipids, 123 POPS lipids, 139 POPE lipids and 121 cholesterol molecules in the inner leaflet.

**Table 1:** Lipid bilayers used in the simulations

Bilayer model	Lipid composition of the outer leaflet	Lipid composition of the inner leaflet	Size (nm^2^)
Asymm	PC/SM/CHOL (~1:1:1)	PC/PE/PS/CHOL (~35 %/25 %/20 %/20 %)	~19 × 19
Symm_O	PC/SM/CHOL (~1:1:1)	PC/SM/CHOL (~1:1:1)	~19 × 19
Symm_I	PC/PE/PS/CHOL (~35 %/25 %/20 %/20 %)	PC/PE/PS/CHOL (~35 %/25 %/20 %/20 %)	~19 × 19

### Coarse-Grained MD Simulations

Coarse-grained (CG-MD) simulations were performed using the MARTINI (v2.1) force field (Monticelli et al. 2008) with an elastic network applied to all backbone particles within a cut-off distance of 7 Å. After the protein conversion to coarse-grained representation the protein was inserted in the different bilayers as described in Table 2. The system was solvated and neutralized, energy minimized for 200 steps, and equilibrated for 5 ns with the protein backbone particle restrained. After that, extended simulations up to 10 μs each were run (see Table 1 for more details).

**Table 2:** Summary of the simulations

Simulation	Protein	Bilayer	Duration (µs)
int_tal_O	F2–F3 + α/β* TM + ectodomain	Symm_O	10
int_tal_I	F2–F3 + α/β* TM + ectodomain	Symm_I	10
int_tal_A	F2–F3 + α/β* TM + ectodomain	Asymm	10
tal_αβ_O	F2–F3 + α/β* TM	Symm_O	10
tal_αβ_I	F2–F3 + α/β* TM	Symm_I	10
tal_αβ_A	F2–F3 + α/β* TM	Asymm	10

*TM* transmembrane helix; β* indicates a chimeric β chain (β3/β1D; see main text for details)

All simulations were performed using GROMACS 4.5.1 (www.gromacs.org) (Hess et al. 2008). A Berendsen thermostat (Berendsen et al. 1984) (coupling constant of 1.0 ps; reference temperature 310 K) and barostat (coupling constant of 1.0 ps, compressibility value of 5.0 × 10^−6^ bar^−1^, reference pressure 1 bar) were used. The integration time step was 10 fs. Lennard-Jones and Coulombic interactions were shifted to zero between 9 and 12 Å, and 0 and 12 Å, respectively. The analysis was performed using GROMACS (Hess et al. 2008; van der Spoel et al. 2005), MDanalysis (Michaud-Agrawal et al. 2011) and locally written codes. The water was not polarizable. The lipid diffusion coefficient was calculated as described in (Goose and Sansom 2013). We note that in the diffusion plots 0 represents the diffusion of the lipids in the first annulus (0–10 Å). Lipid spatial densities were normalized by dividing the lipid density by the bin area, the number of frames and the number of lipids in the corresponding bilayer leaflet. Note that for the normalization of the cholesterol densities, because of the cholesterol flip-flop during the simulations, we have used the number of cholesterol in each leaflet from the last frame of each simulation. To estimate errors, diffusion coefficients for protein/lipid interactions were calculated for 5 × 2 μs sub-trajectories derived from each trajectory. The errors in Supplementary Fig. S3 were calculated as the standard deviations of the normalized contacts of the 3 different simulation systems (see Table 1).

## Results

Lipids are distributed asymmetrically between the two leaflets of the plasma membrane: the outer leaflet is rich in sphingomyelin, phosphatidylcholine and cholesterol, whilst the inner leaflet contains mainly phospholipids and cholesterol (van Meer et al. 2008). In order to study the behaviour of the integrin/talin complex in a model of a plasma membrane, an asymmetric bilayer was constructed (see Table 1) into which the integrin/talin complex was inserted.

The integrin/talin complex (Table 2) was embedded into the asymmetric lipid bilayer such that the integrin TM domain (2K9J in Fig. 1) spanned the bilayer with the αIIb TM helix parallel to the bilayer normal (Lau et al. 2009; Yang et al. 2009), whilst the β TM helix adopted a tilted (~30°) orientation relative to the bilayer normal. Thus the positively charged surface patch of the talin F2 subdomain was situated at the surface of the inner leaflet of the bilayer. In the *tal_αβ* systems (Table 2) the ectodomain (3IJE in Fig. 1) was removed, in order to enable us to examine the intrinsic dynamics of the talin F2–F3/αβ TM complex (3G9W/2K9J in Fig. 1) and of its interactions with the bilayer in the absence of possible modulation by the ectodomain. Following protein insertion in the bilayer, CG-MD simulations were performed for each system. For comparison, simulations were also performed in the bilayers with similar compositions as either the outer or inner leaflet of the asymmetric bilayer (Tables 1 and 2).

### Integrin/Talin Complex Dynamics

**Fig. 2:**
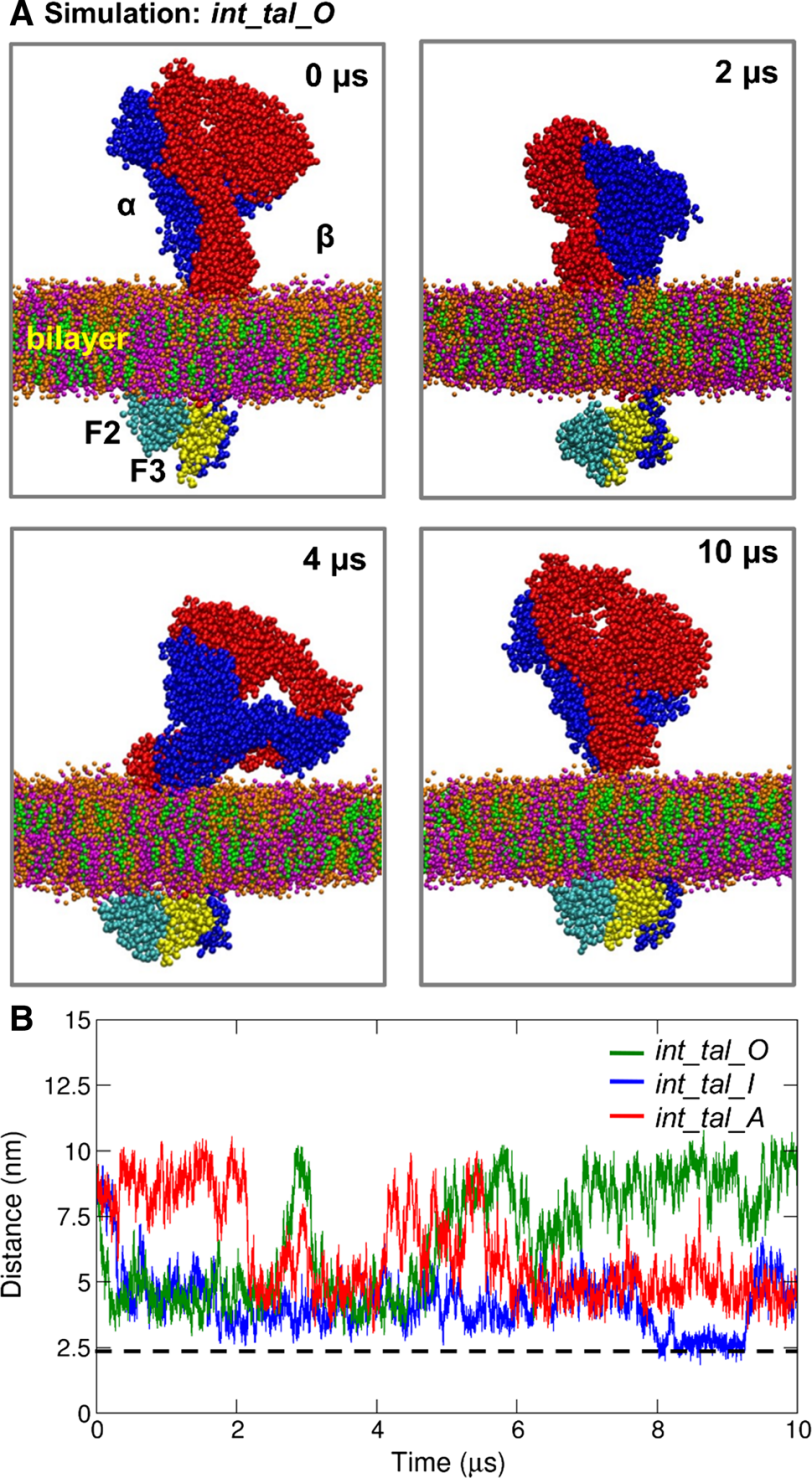
**a** Progress of the *int_tal_O* simulation. Snapshots of the simulation are shown at 0, 2, 4 and 10 μs. The integrin α subunit is shown in *red*, the β subunit in *blue*, the talin F2 domain in cyan and the F3 domain in *yellow*. The bilayer lipids are shown in *magenta* for POPC, *orange* for SM and *green* for cholesterol. The waters and counter ions are omitted for clarity. **b** Distance (*z* component) between the centre of mass of residues 727–735 (of the Calf-1 domain) and the centre of mass of the bilayer for the *int_tal_O* (*green*)*, int_tal_I* (blue) *and int_tal_A* (*red*) simulations. The *black-dotted line* indicates the position of the lipid phosphates (Color figure online)

**Fig. 3:**
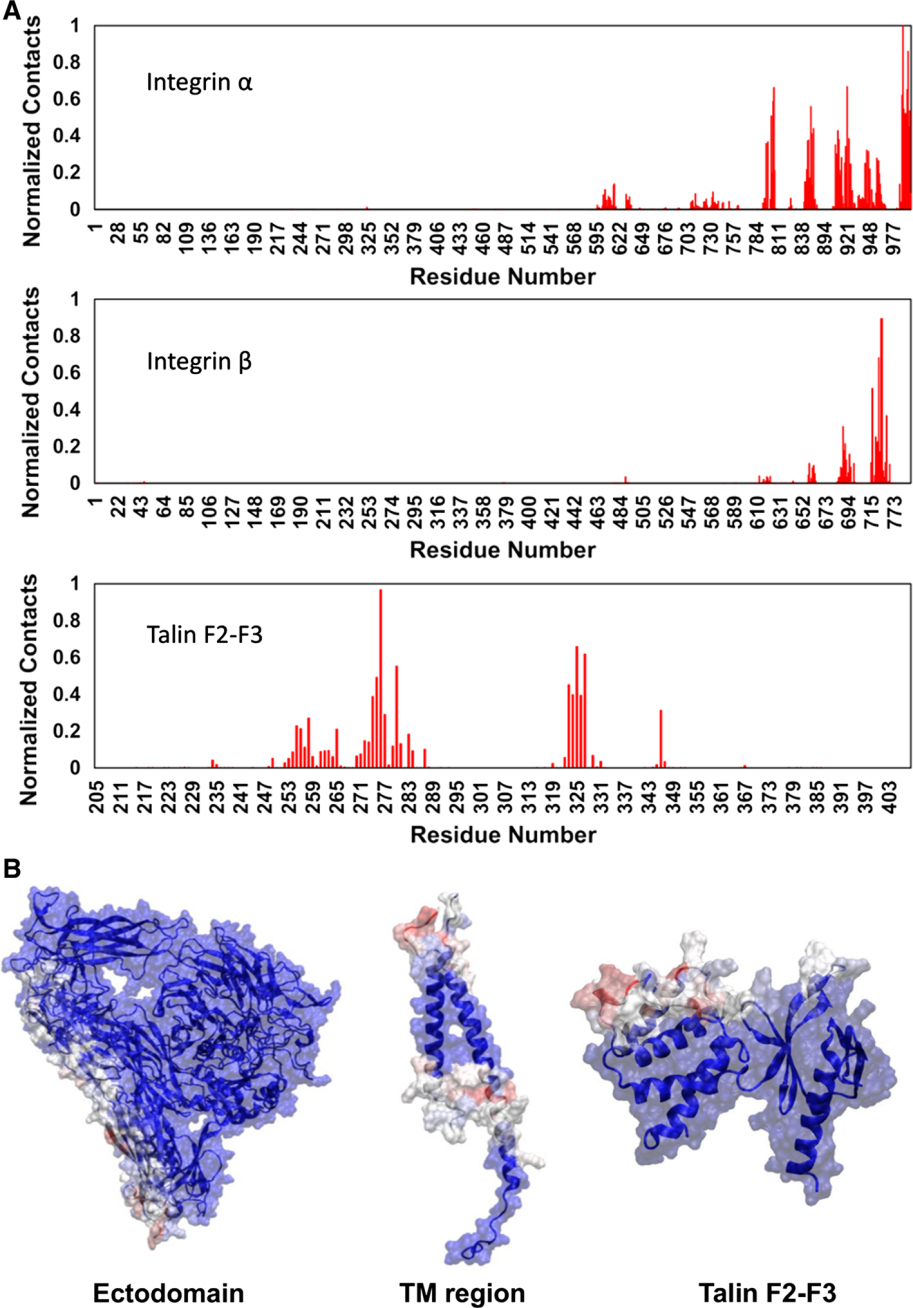
**a**, **b** Interactions of the integrin/F2–F3 complex with lipids. **a** The normalized number of interactions of the integrin/F2–F3 complex with the POPS lipids in the *int_tal_I* simulation are shown. **b** The contacts are mapped onto the integrin/F2–F3 complex structures. *Blue* represent no/low number of contacts, *white* medium number of contacts and *red* high number of contacts. The contacts are shown separately for the integrin ectodomain, the integrin TM region and the talin F2–F3 domains for clarity. The normalization was done using the highest number of contacts from the whole integrin/F2–F3 complex (Color figure online)

Inspection of the motion of the integrin receptor revealed substantial fluctuations of the ectodomain in the simulations due to the flexible linkers between the TM region and the ectodomain (Fig. 2). Despite the usage of an elastic network model (ENM) network to model the protein secondary and tertiary structure (see Methods), no ENM restrictions were present in the flexible loop region connecting the structured parts of the TM domains and the ectodomains. Examination of the contacts between the integrin ectodomain and the lipids in the bilayer demonstrated that in the *int_tal_O* and *int_tal_A* simulations (i.e. in the absence of any negatively charged lipids in the outer leaflet in our simulations) no significant direct ectodomain/bilayer interactions occurred. It should be noted, however, that negatively charged lipids e.g. GM1 and GM3, which may regulate integrin signalling (Pande 2000), are also found in the outer leaflet of the plasma membrane and may facilitate interactions between the ectodomain and the bilayer (Pande 2000). Glycosphingolipids were also shown to regulate signalling by other signalling receptors e.g. EGFR (Coskun et al. 2011; Kawashima et al. 2009). In contrast in the *int_tal_I* simulation, analysis of the ectodomain movement revealed transient interactions between the lower leg part (but not the upper legs) of the ectodomain (i.e. Calf-1 and Calf-2 domains on the α subunit) and the outer leaflet of the bilayer, mainly with the headgroups of PS and PE (Figs. 2, 3). In particular residues K605, K615, K616 and K733 on Calf-1 and K802, N804, N805, Q878 and K921 on Calf-2 made the largest number of contacts with POPS lipids. Due to the unstructured regions between the ectodomain and the TM region, the lower parts of the Calf-2 domain on the α subunit and of the β-tail domain on the β subunit are in contact with the bilayer in all simulation systems.

It should be noted that because of the use of an ENM to maintain the secondary and tertiary structure in the CG model, substantive conformational changes were not possible within the integrin receptor, and thus the integrin receptor remains “locked” in its inactive state. Thus, the observed contacts between the ectodomain and bilayer lipids indicate that in the plasma membrane the integrin ectodomain is very dynamic even when remaining in an inactive state. This is expected to have functional implications because possible stable interactions with lipids may mask some ligand-binding sides on the receptor. The behaviour observed in our simulations is consistent with studies of integrins reconstituted into nanodiscs which demonstrated that in the active state the integrin ectodomain adopts an extended form almost perpendicular to the membrane (Ye et al. 2010). Interestingly, experimental and computational studies on the epidermal growth factor receptor (perhaps the best studied example of the receptor tyrosine kinase family of receptors) demonstrated that its ectodomain may also interact with the extracellular leaflet of the membrane and that these interactions may regulate its function (Abd Halim et al. 2015; Arkhipov et al. 2013). A recent study has also shown that *N*-glycosylation of the ectodomain alters the dynamic behaviour of the EGFR/membrane complex (Kaszuba et al. 2015).

Analysis of the contacts of the F2–F3 domain pair with lipids in the *int_tal_O* simulation suggested transient interactions of the positively charged patch (res: 255–285) of talin F2 with the bilayer (Fig. 2). From previous work (Anthis et al. 2009; Kalli et al. 2010, 2013a), we suggest that these interactions were transient because there were no negatively charged lipids in the membrane to stabilize the talin F2/lipid interactions. In the *int_tal_I* and *int_tal_A* simulations, the presence of negatively charged lipids stabilized the interactions between the cytoplasmic protein and the bilayer. The talin F3 subdomain interacts with the membrane via a positively charged loop (res: 320–328). In all the simulations with the F2–F3/αβ complex the interactions of the talin F2–F3 pair with the bilayers were similar to the interactions observed in the simulations with the complete integrin receptor (above). In particular, when the F2–F3/αβ complex was inserted in the *Symm_O*, transient interactions of the F2 subdomain with the bilayer have been observed. The F2/bilayer complex was stabilized when the *Symm_I* and the *Asymm* bilayers were used due to the strong interactions of positively charged residues on the F2 subdomains with the PS lipids in the bilayer. Merging the interactions of talin F2 domain with the POPS lipids from the *int_tal_I* and *int_tal_A* simulations reveals that residues K274 to R276 and K280 on F2 made the largest number of interactions (Supporting Information Fig. S3).

### Lipid Organization Around the Integrin/Talin Complex

**Fig. 4:**
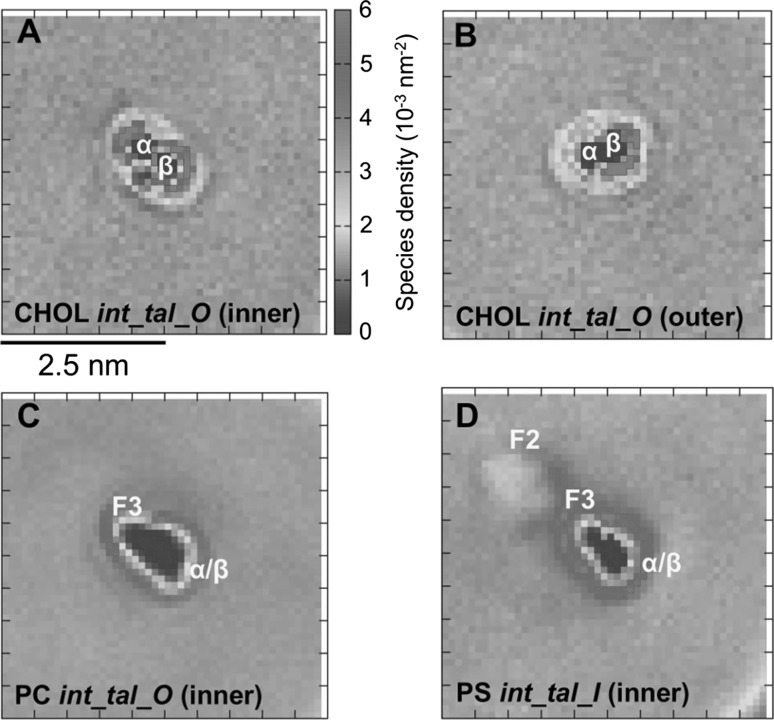
Two-dimensional average lipid headgroup densities for the cholesterol molecules from the *int_tal_O* simulation (**a**, **b**) and for the POPC lipids from the *int_tal_O* simulation (**c**) and POPS lipids from the *int_tal_I* simulation (**d**) around the integrin receptor. The *diagram* shows the probability of finding the lipid at a given point in the bilayer plane. *Blue* represents low probability through *red* for high probability. In the *int_tal_I* simulation (**d**) a second high density region was observed which reflects the strong interactions of the F2 domains with the lipids (Color figure online)

In order to study in more detail the organization and interactions of the different lipid types around the integrin receptor we calculated two-dimensional average lipid headgroup densities around the integrin receptor (Fig. 4 and Figs S1, S2). For the *int_tal_O* system this analysis revealed an asymmetric distribution of lipids between the two leaflets with a high density ring of PC lipids around the integrin TM region in the inner leaflet. The same analysis revealed a homogenous distribution for sphingomyelin (SM) in both leaflets and a high density of cholesterol (CHOL) close to the TM region. Examination of the sequence of the integrin αIIbβ3 TM region used in this study reveals that neither of the two subunits contains a tyrosine residue in the TM region. Tyrosine is a key residue in the CRAC motif ((L/V)-X_1−5_-(Y)-X_1−5_-(K/R)) which has been suggested to interact with cholesterol (Fantini and Barrantes 2013). The αIIb TM region does contain a V^990^GFFKR^995^ sequence on the cytosolic side of the membrane which might be thought to form a cholesterol-interaction motif (Fantini and Barrantes 2013). However, this sequence is located at the lipid/water interface with the two phenylalanine residues pointing towards the β3 TM helix, and so elevated cholesterol density was not observed in the vicinity of this sequence. Cholesterol was not uniformly distributed around the TM region of the protein. Residues 966 and 969 in the extracellular region of the αIIb subunit and residues 985, 986, 989 and 990 in the cytosolic region of the αIIb subunit made the highest number of contacts with cholesterol ROH group. Similarly, residues 695, 696 and 699 in the β1/β3 TM extracellular regions and residues 756 and 757 in the β1/β3 TM cytosolic regions made significant contacts with cholesterol (see Figs. 4 and Supporting Information S4 and S5). A similar asymmetric distribution of lipid interactions between the two leaflets was also observed in the *int_tal_I*. Interestingly, the high density ring of PC lipids around the integrin TM region in the inner leaflet (in the *int_tal_O* simulation) was replaced by a similar high density ring of PS lipids in the *int_tal_I* simulation. This ring was uniformly distributed around the TM region indicating that the PS distribution is affected not only by the F3 acidic loop which is close to the TM region but also by the positively charged residues in the integrin TM region (i.e. αIIb K989, K994, R995 and R997 and β1/β3 K716, H758, R760, R761 and K765 residues). The stronger and more stable interactions of the F2 domain with the bilayer in this simulation (see above) resulted in a second high density of PS lipids around the F2 domain, which was not present in the *int_tal_O* simulation. The distributions of cholesterol in the *int_tal_O* and *int_tal_I* simulations were very similar (Supporting Information Figs. S1, S2 and S5). Analysis of possible variations in the interactions of cholesterol and of POPS molecules with the integrin/F2–F3 complex obtained by dividing our trajectories in 5 sub-trajectories (i.e. five consecutive sub-trajectories each of 2 μs duration) suggests that the cholesterol and POPS interactions were retained during the whole of the overall trajectory.

Comparison of the two-dimensional lipid headgroup densities of the outer and the inner leaflets of the asymmetric bilayer in simulation *int_tal_A* with the same leaflets in the *int_tal_O* and *int_tal_I* simulations (i.e. the outer leaflet of the *int_tal_A* simulation was compared with the outer leaflet of the *int_tal_O* simulation and the inner leaflet of the *int_tal_A* simulation was compared with the inner leaflet of the *int_tal_I* simulation) revealed similar distribution for all lipids (Figs. S1 and S2). Therefore, the organization of each leaflet in the asymmetric bilayer closely resembled that of the equivalent leaflet in the two simulations described above.

**Fig. 5:**
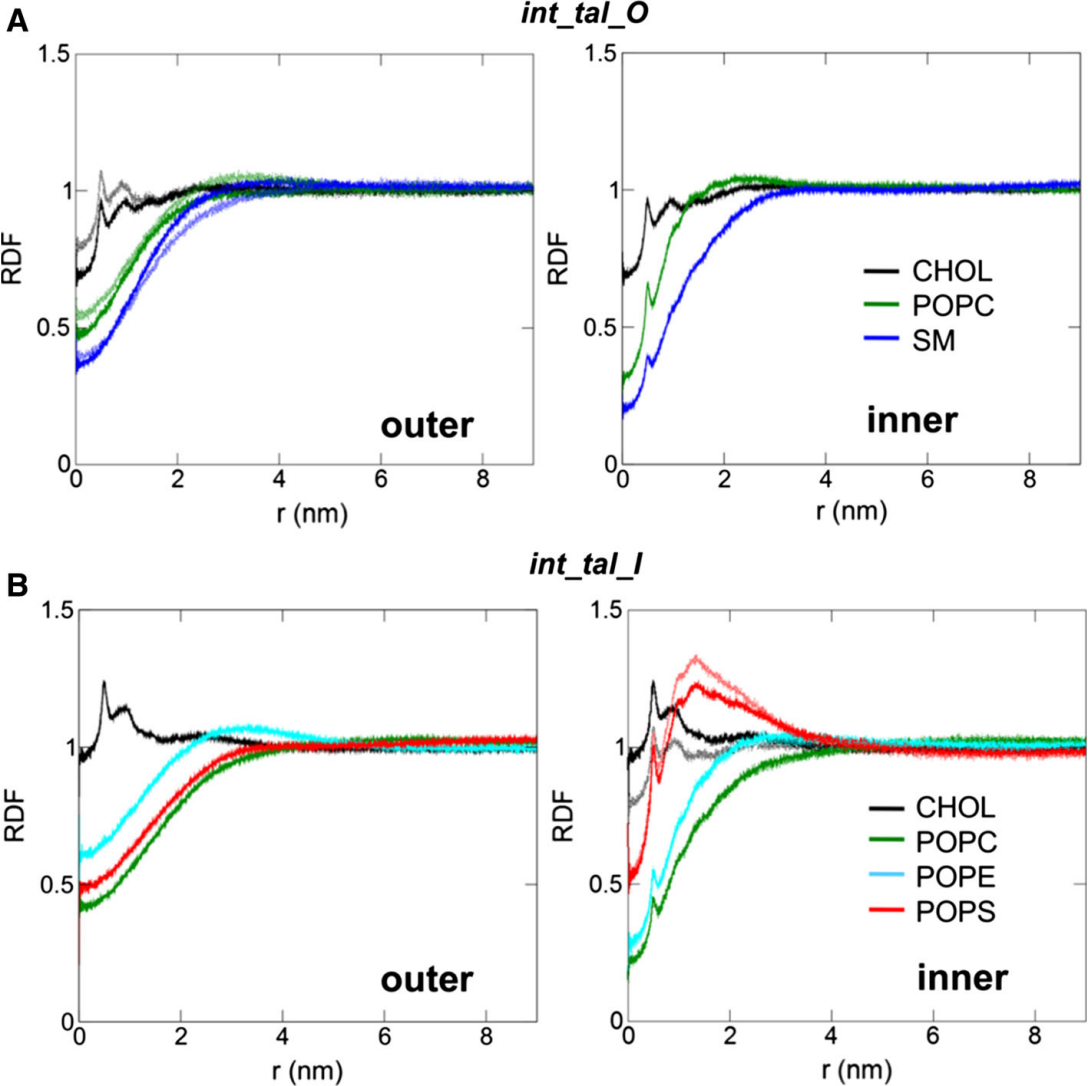
**a**, **b** The lipid radial distribution function calculated in the *xy* (i.e. bilayer) plane for the *int_tal_O* (**a**) and the *int_tal_I* (**b**) simulations. The distribution for each lipid type is shown separately for the outer (*left*) and the inner (*right*) leaflet with the exception of cholesterol for which we show the lipid radial distribution for the whole bilayer. This was done to take into account the cholesterol flip-flop during the simulations. The radial distribution was calculated using the phosphate atoms of the lipids around the integrin TM regions. The *dotted lines* in the outer leaflet of the *int_tal_O* simulation (**a**) and in the inner leaflet of the *int_tal_I* simulation (**b**) represent the radial distribution function for lipids in the outer (**a**) and inner (**b**) leaflets of the *int_tal_A* simulation

To further quantify the distributions of lipids around the integrin TM region, radial distribution functions (RDFs) of the lipids relative to the protein TM domain were calculated (Fig. 5). For the outer leaflet in the *int_tal_O* simulation, a higher density was observed for cholesterol indicating its preference to accumulate close to the TM region. The density (i.e. close to the integrin TM region) was lower for PC and for SM suggesting a preferred pattern of lipid distribution close to the integrin TM region in the outer leaflet, i.e. CHOL > PC > SM. In the inner leaflet the density for the PC was lower close to the TM region compared to the cholesterol, however high density for the PC was observed at ~2 nm, corresponding to the location of the talin F2–F3 domain. This indicates that despite the interactions between the talin F2 domain (above) and the lipids being only transient, the PC distribution was affected (Fig. 5a).

In the *int_tal_I* simulation for which a different bilayer (i.e. PC/PE/PS/CHOL) was used, in the outer leaflet the high density of cholesterol around the integrin TM region was accompanied by the depletion of all three phospholipids (PC, PE and PS). The density of the POPE lipids was somewhat higher compared to POPC and POPS lipids. This lipid distribution pattern was modified in the inner leaflet since the density of negatively charged lipids (PS) at ~2 nm was higher compared to the cholesterol density (Fig. 5b). The high density of PS lipids was a result of interactions with the positively charged loop in the talin F3 subdomain which was situated close to the integrin TM region (i.e. it interacted with the TM domain) and the cluster of positively charged residues in the integrin TM region. The high density of PS lipids in the inner leaflet (which was higher compared to the PC/SM density at the same area in the *int_tal_O* simulation) suggests a strong preference for the cytoplasmic protein to interact with negatively charged PS lipids.

Comparison of the lipid radial distribution function observed in the simulation with the asymmetric bilayer (*int_tal_A*) with those observed in the *int_tal_O* and *int_tal_I* simulations revealed very similar distributions for all lipid types in both leaflets (Fig. 5 and S1 and S2). This augments the previous observation that the lipid organization of each leaflet in the asymmetric bilayer is comparable to that of the equivalent leaflet in the *int_tal_O* and *int_tal_I* simulations described above. In all cases the lipid distributions were also similar for the simulations with the F2–F3/αβ complex reflecting the fact that no direct stable interactions between the lipids and the ectodomain occurred.

### The Integrin/Talin Complex Alters Lipids Mobility

**Fig. 6:**
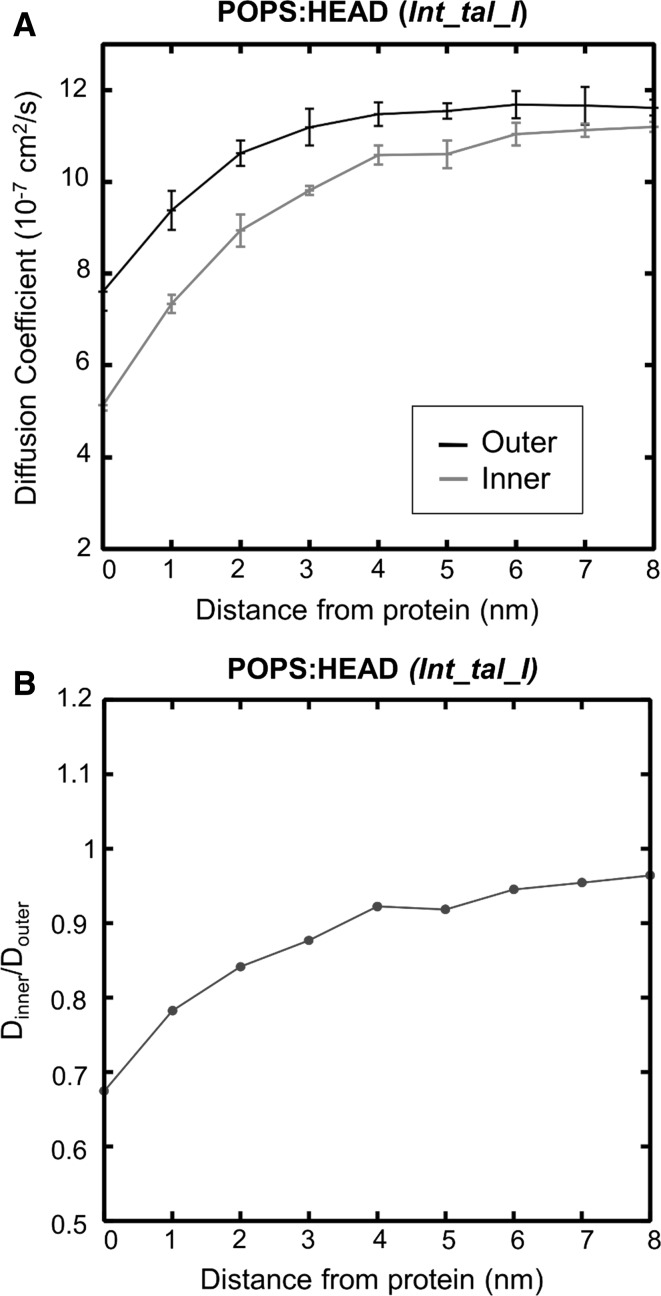
**a**, **b** Diffusion coefficient (*D*) of the head group of POPS lipids in the outer and the inner leaflet of the *int_tal_I* (**a**) simulation as a function of distance from the protein centre of mass. **b** Ratio of the aforementioned diffusion coefficients. In the figure we show only the curve for timescale (Δ*t*) 6 ns. The errors were calculated as the standard deviations of five sub-trajectories (5 × 2 μs)

The local clustering of lipids resulting from their interactions with the integrin/talin complex (see above) is anticipated to alter lipid mobility. To examine this further, the lipid lateral diffusion coefficient (*D*) in the two leaflets separately was calculated (as described in (Goose and Sansom 2013)). In this calculation the lateral diffusion coefficient was estimated as a function of timescale (Δ*t*) to capture the different modes of diffusion (i.e. those dominating at shorter timescales and those dominating at longer timescales). At larger timescales (Δ*t*), this calculation is expected to yield converging values for the lateral diffusion coefficient (*D*). Overall, this calculation revealed a value of ~10^−7^ cm^2^/s for lipid components in all the simulations. Significantly, calculation of the diffusion coefficient relative to the radial (i.e. lateral) distance from the protein complex revealed a reduced diffusion coefficient for lipids in a layer immediately around the protein, suggesting a slowing-down effect of protein contacts on the lipids. The effect was strongest for the region within 1 nm around the protein. For distances greater than *ca*. 5 nm from the protein the diffusion coefficient reached a plateau (Fig. 6).

To characterize this effect more quantitatively, the space around the protein was divided into annuli of width 1 nm each and the diffusion coefficient was measured in each annulus (Supporting Information Fig. S6). For example, for the inner leaflet of the *int_tal_O* simulation this analysis showed that the diffusion coefficient of the PC headgroups was ~5 × 10^−7^ cm^2^/s in the first annulus (0–1 nm from the protein), ~6.5 × 10^−7^ cm^2^/s in the second annulus (1–2 nm) and ~8.5 × 10^−7^ cm^2^/s in the outer annulus (8–9 nm) and the SM diffusion coefficient was ~6 × 10^−7^ cm^2^/s (0–1 nm), ~7 × 10^−7^ cm^2^/s (1–2 nm) and ~9 × 10^−7^ cm^2^/s (8–9 nm) demonstrating a slowing-down effect in the vicinity of the protein for the lipid species in the system. Similar patterns of locally depressed lipid diffusion coefficients around the protein were observed in the *int_tal_I* and *int_tal_A* simulations. For cholesterol we observed a similar slowing-down effect, complicated in part by flip-flop of cholesterol between the two leaflets.

**Fig. 7:**
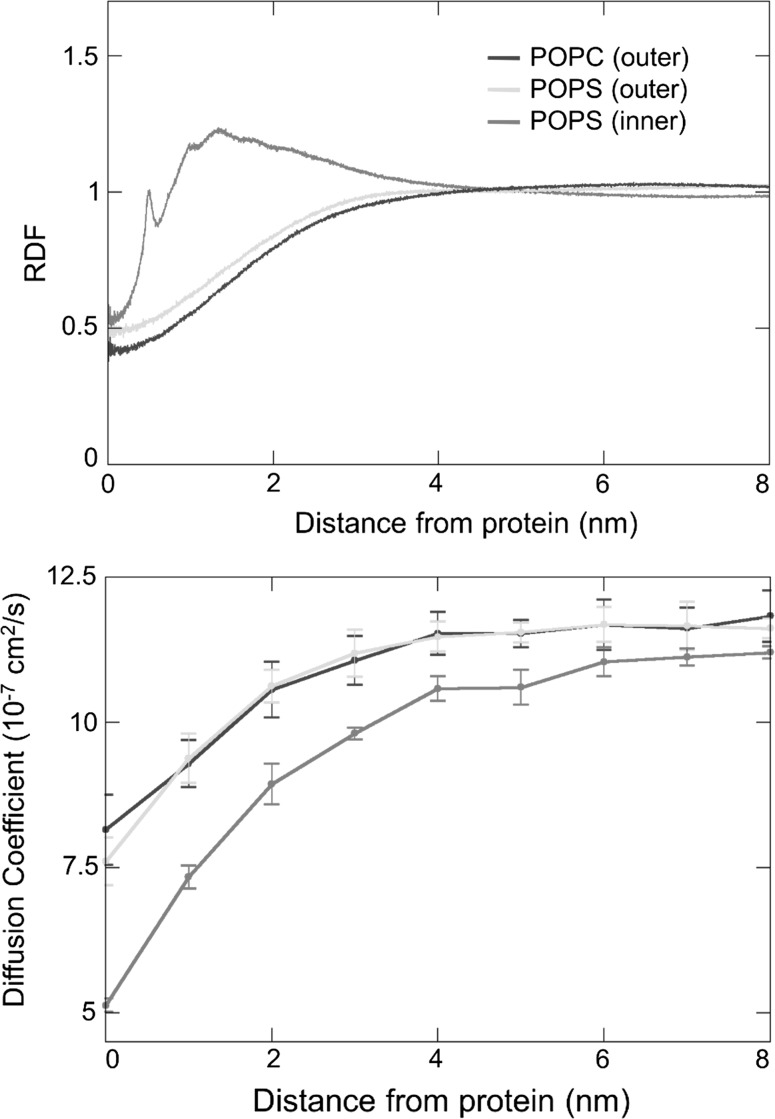
The lipid radial distribution function calculated in the *xy* (i.e. bilayer) plane for the POPC (*blue*) and POPS (inner leaflet-*red*, outer leaflet-*green*) lipids from the *int_tal_I* simulation. The diffusion coefficient of the headgroups of the same lipids is shown as a function of distance from the protein. The errors were calculated as the standard deviations of five sub-trajectories (5 × 2 μs) (Color figure online)

The lipid mobility (i.e. diffusion coefficient) profiles of each simulation system correlate well with the equilibrium distributions (i.e. radial distribution functions) of lipids around the integrin/talin complex. Thus, those lipid types whose radial distribution profiles indicated strong association with the protein had a lower mobility compared to other lipids. This can be seen for example if one compares PS in the inner leaflet (where it shows a high RDF density) with the outer leaflet (where the first RDF density is low). For PS *D*_0−1nm_ for the inner leaflet is ~70 % that for the outer leaflet. PS lipids, which had a high density in its radial distribution function also had a lower diffusion coefficient in the 0−1 nm region compared to those lipids e.g. PC which did not show such high density in their radial distribution (Fig. 7). Thus for the *int_tal_I* simulation for PS the *D*_0–1nm_ */D*_*bulk*_ ratios is ~0.5, whereas for PC the ratio is ~0.65.

## Discussion

**Fig. 8:**
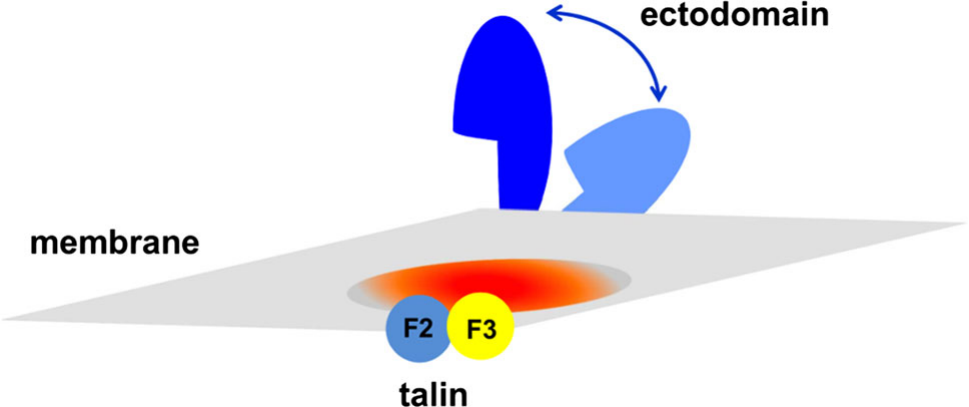
Model of the complete integrin receptor in a plasma membrane. The ectodomain is shown in *orange*, the membrane in *grey*, the talin F2 domain in *cyan* and the talin F3 domain in *yellow*. The *red* region represents the region around the protein (up to ~30 Å) for which the protein induces a slowing down of lipids (Color figure online)

In this study extended simulations were used to probe the dynamics and lipid interactions of an integrin/talin complex in complex bilayers that mimic cell membranes. The results illustrate the dynamic nature of the integrin ectodomain and demonstrate the prominent role of the integrin/lipid interactions in membrane leaflet structure and dynamics. In particular, we observe a significant slowing-down effect in the vicinity of the protein for those lipids which form interactions with the protein complexes (Fig. 8).

The first outcome of this study is that the presence of the integrin/talin complex modifies the local lipid profile in all types of bilayers examined. In the simulations, depending on the nature/strength of the protein/lipid interactions the organization of the lipids in each leaflet was altered to accommodate the interactions with the protein domains. This change in the lipid profile resulted in an asymmetric distribution of lipids between the leaflets of the bilayer. Experimental studies have suggested that other membrane proteins such as rhodopsin induced reorganization of phospholipids in rod disc membranes (Hessel et al. 2001). Lipid reorganization was also observed after the binding of membrane-active peptides to the membrane (Joanne et al. 2009), around TM helices (Kaiser et al. 2011; Koldsø and Sansom 2012) and after binding of peripheral proteins (Kalli et al. 2014; Kalli et al. 2013b; Kalli and Sansom 2014). Additionally, computational approaches suggested reorientation of lipids in one leaflet upon binding of nanosubstrates (Varma et al. 2012) to the lipid bilayer. The asymmetric distribution of the two leaflets is expected to have implications in biological processes (Balasubramanian and Schroit 2003) since previous studies have shown that leaflet asymmetry is important for the regulation of permeability in the membrane (Negrete et al. 1996) and for membrane stability (Manno et al. 2002).

The lipid diffusion rate in all simulations was ~10^−7^ cm^2^/s. The various protein/lipid interactions, however, limited the motion of the lipids in an annulus of ~2.5 nm around the protein suggesting a slowing-down effect for lipids in the vicinity of the protein. Lipids around a protein often are referred to as “annular” lipids. The concept of a lipid annulus around membrane proteins is supported by electron paramagnetic resonance (EPR) studies with spin-labelled lipids where it was possible to detect slower mobility of lipids in membranes with protein domains (Lee 2003, 2011; Marsh 2010). This concept is further supported by simulation studies (Niemela et al. 2010) and by crystal structures obtained with lipids bound on the protein surface (Gonen et al. 2005; Luecke et al. 1999). Despite the fact that the TM dimer surface is relatively smooth, the effect observed in the simulations here was relatively strong. Larger proteins or proteins with well-defined binding surfaces might further enhance this phenomenon (Niemela et al. 2010). It has been suggested that integral membrane proteins and their adjacent lipids form transient complexes that move in a concerted manner in the membrane (Niemela et al. 2010). This, in combination with the fact that membranes are highly crowded (~30 to 40 % proteins) (Dupuy and Engelman 2008; Ryan et al. 1988) suggests that the number of free lipids in cell membranes is limited due to the formation of various protein/lipids complexes.

Examination of the lipid distributions suggests an elevated density of cholesterol close to the integrin TM region in all bilayers. This is in good agreement with experimental results which suggested that the presence of integrins affects the cholesterol distribution (Pankov et al. 2005). Indeed, cholesterol has been suggested to interact with and possibly regulate a number of types of receptor (Hamouda et al. 2005; Hanson et al. 2008; Lingwood et al. 2011; Sahu et al. 2012; Shinoda et al. 2009). Our simulations confirm the electrostatic nature of the talin/bilayer interactions. Indeed, in the simulations the talin F2 subdomain interacted strongly with the bilayer (i.e. not transiently) only when negatively charged PS lipids were present in the inner leaflet. This observation is consistent with experimental results (Anthis et al. 2009) which suggest that negatively charged moieties are crucial for the talin/membrane interactions and thus for integrin activation. Interestingly, it has been shown recently using both experimental and simulation techniques that annular anionic lipids e.g. PS or PG stabilize the integrin αIIbβ3 TM region (Schmidt et al. 2015). It should also be noted that experimental studies suggested that talin/lipid electrostatic interactions are regulated by the net negative charge of the membrane and not the lipid type (i.e. POPS, PIP_2_) involved (Elliott et al. 2010). However, it has been demonstrated that talin head domain/PIP_2_ interactions promote integrin clustering (Saltel et al. 2009). PIP molecules may also be expected to regulate the association of kindlins with the cell membrane and with integrins, given that kindlins contain a PH domain, frequently associated with PIP binding (Yates et al. 2012). Kindlins act in synergy with talin to induce integrin activation (Anthis and Campbell 2011). Kindlins are expected to have similar structure to talin, but with an additional PH domain and a longer loop on the F1 domain. However, no structures of the kindlin head domain, with the exception of the PH domain, have been solved. Additionally, experimental studies using full-length αIIbβ3 integrins in phospholipid nanodiscs showed that talin/phospholipid interactions are needed for the talin-mediated integrin inside-out activation of unclustered integrin (Ye et al. 2010). The simulations also demonstrate the dynamic nature of the integrin ectodomain even whilst it remains in the inactive state. The flexible linker between the TM region and the integrin ectodomain allows large fluctuations of the ectodomain. The ectodomain only interacted transiently with the bilayer in the presence of negatively charged lipids.

It is important to also consider some limitations of this study. The first limitation is the use of coarse-grained molecular dynamics simulations. This results in the approximation of the electrostatic and H-bonds interactions in the protein–lipid and lipid–lipid interactions, however it has been shown that CG simulations can rather accurately predict the interactions of PIP_2_ molecules (Stansfeld et al. 2009) and cardiolipins (Arnarez et al. 2013) with integral membrane proteins. A perhaps more important limitation is the use of an elastic network (ENM) to maintain the secondary and tertiary structure of the protein. Future studies of the integrin receptor are needed using (computationally demanding) atomistic simulations which will allow us to explore more fully the conformational dynamics of the receptor in the bilayer. Additionally, in our integrin/talin model we have only included the talin F2–F3 domain pair, rather than a complete talin head domain. However, it is known that the talin F2–F3 pair alone is sufficient to activate integrins (Anthis et al. 2009; Calderwood et al. 2002).

In summary, this study demonstrates the dynamic nature of the integrin receptor and suggests that its presence has a significant effect on the structure and dynamics of membrane lipids. Recent studies of all the members of another significant family of signalling receptors i.e. Receptor Tyrosine Kinases (Lemmon and Schlessinger 2010; Wang et al. 2009) which exhibit similar topology to the integrin receptor (i.e. with a dimeric TM region and a negatively charged cytosolic site) also showed a rearrangement of the local lipid environment around the TM region mainly due to the interaction of negatively charged residues with anionic lipids (Hedger et al. 2015). Therefore, our results concerning the effect of the integrin receptor on the structure and dynamics of the bilayer leaflets are of particular biological significance because they may be important for signal transduction events.

## Electronic supplementary material

Below is the link to the electronic supplementary material.

Supplementary material 1 (PDF 1335 kb)
