# Supplementary material for: The Integrin Receptor in Biologically Relevant Bilayers: Insights from Molecular Dynamics Simulations
Source: J Membr Biol. 2016 Jul 27;250(4):337–51. doi: 10.1007/s00232-016-9908-z (PMC5579164; doi:10.1007/s00232-016-9908-z)
Supplement: Supplementary file 1 — Supplementary material 1 (PDF 1335 kb) [file 232_2016_9908_MOESM1_ESM.pdf]

**Supporting Information for:**

# The Integrin Receptor in Biologically Relevant Bilayers: Insights from Molecular Dynamics Simulations

*Antreas C. Kalli, Tomasz Rog, Ilpo Vattulainen, Iain D. Campbell & Mark S. P. Sansom*

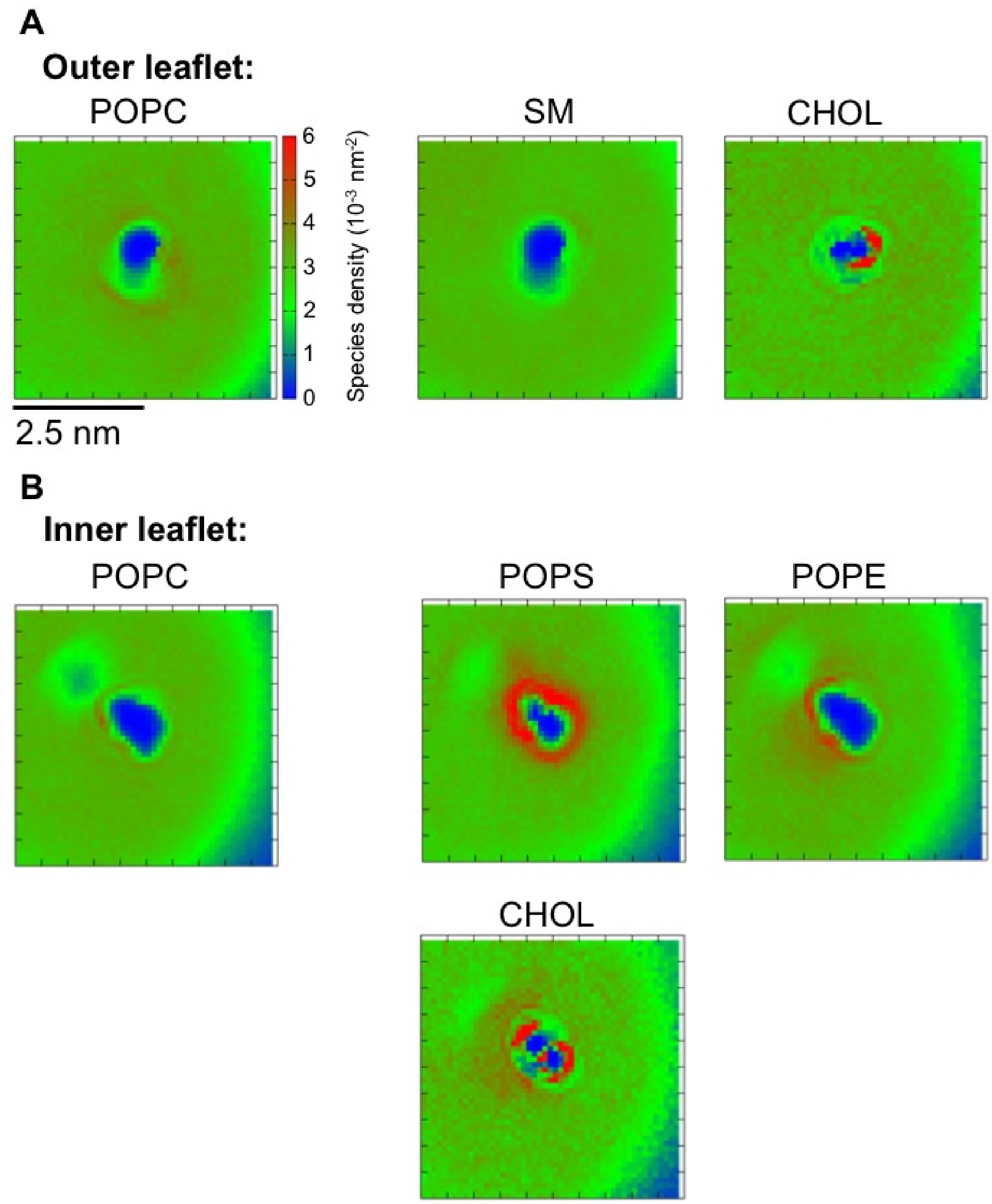


*Supplementary Fig. S1:*
A,B Two-dimensional average lipid headgroup densities around the integrin receptor for the outer (A) and inner (B) leaflets in the *int_tal_A* simulation. The diagram shows the probability of finding the lipid at a given point in the bilayer plane around the protein. Blue represents low probability through red for a high probability.

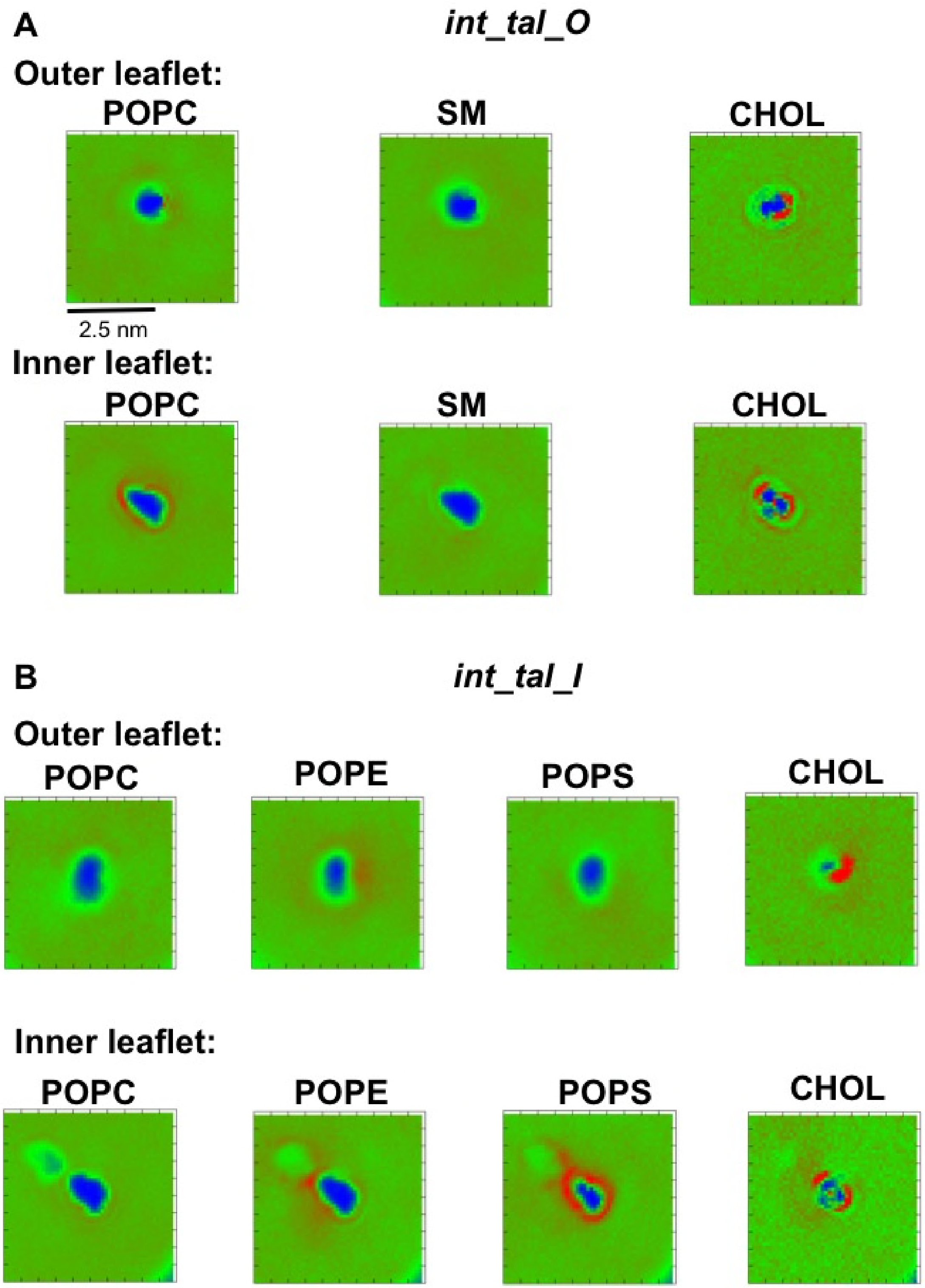


*Supplementary Fig. S2:*
A,B Two-dimensional average lipid headgroup densities around the integrin receptor for the *int_tal_O* (A) and *int_tal_I* (B) simulations. The diagram shows the probability of finding the lipid at a given point in the bilayer plane around the protein. Blue represents low probability through red for a high probability.

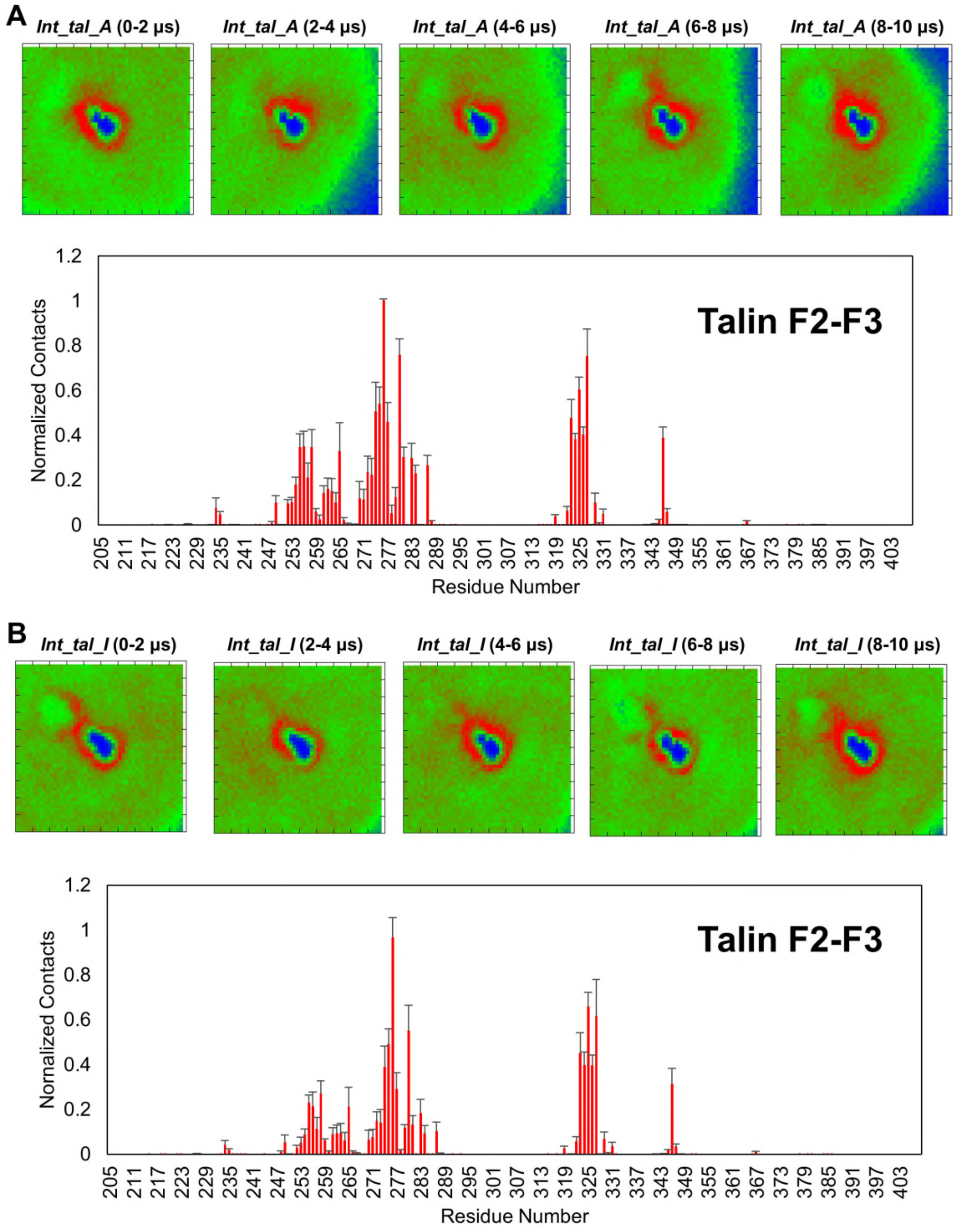


*Supplementary Fig. S3:*

A,B Two-dimensional average lipid densities (of the POPS headgroups) around the integrin/F2-F3 complex for the inner leaflet of the *int_tal_A* (A) and *int_tal_I* (B) simulation systems. The diagram shows the probability of finding the lipid at a given point in the bilayer plane around the protein. Blue represents low probability through red for a high probability. The normalized contacts between the POPS phosphate atoms and the integrin/F2-F3 complex are shown below the densities for the same systems. For clarity, only the talin F2-F3 domain, which made a large number of interactions with POPS molecules, is shown. The errors were calculated as the standard deviations from 5 sub-trajectories (5 x 2 μs). The normalization for the contacts was made as described in Figure 3.

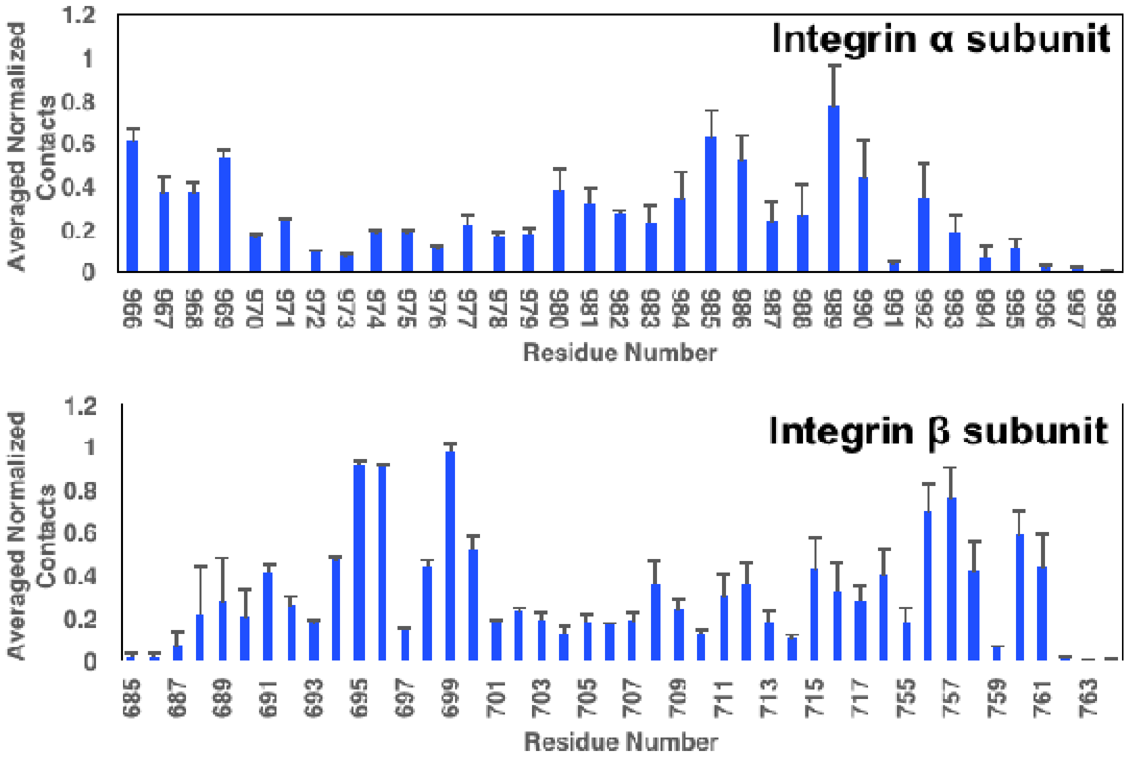


*Supplementary Fig. S4:*

Normalized average number of contacts of the integrin/F2-F3 with cholesterol. For this calculation the contacts between the integrin/F2-F3 complex with the cholesterol ROH group from the 3 different simulation systems were added together (see Table 1). A cut off distance of 0.7 nm was used to define a contact. Only the TM regions of the integrin receptor, which made the largest number of interactions with cholesterol, are shown for clarity. The errors were calculated as the standard deviations of the normalized contacts between cholesterol and the protein in the 3 different simulation systems (see Table 1).

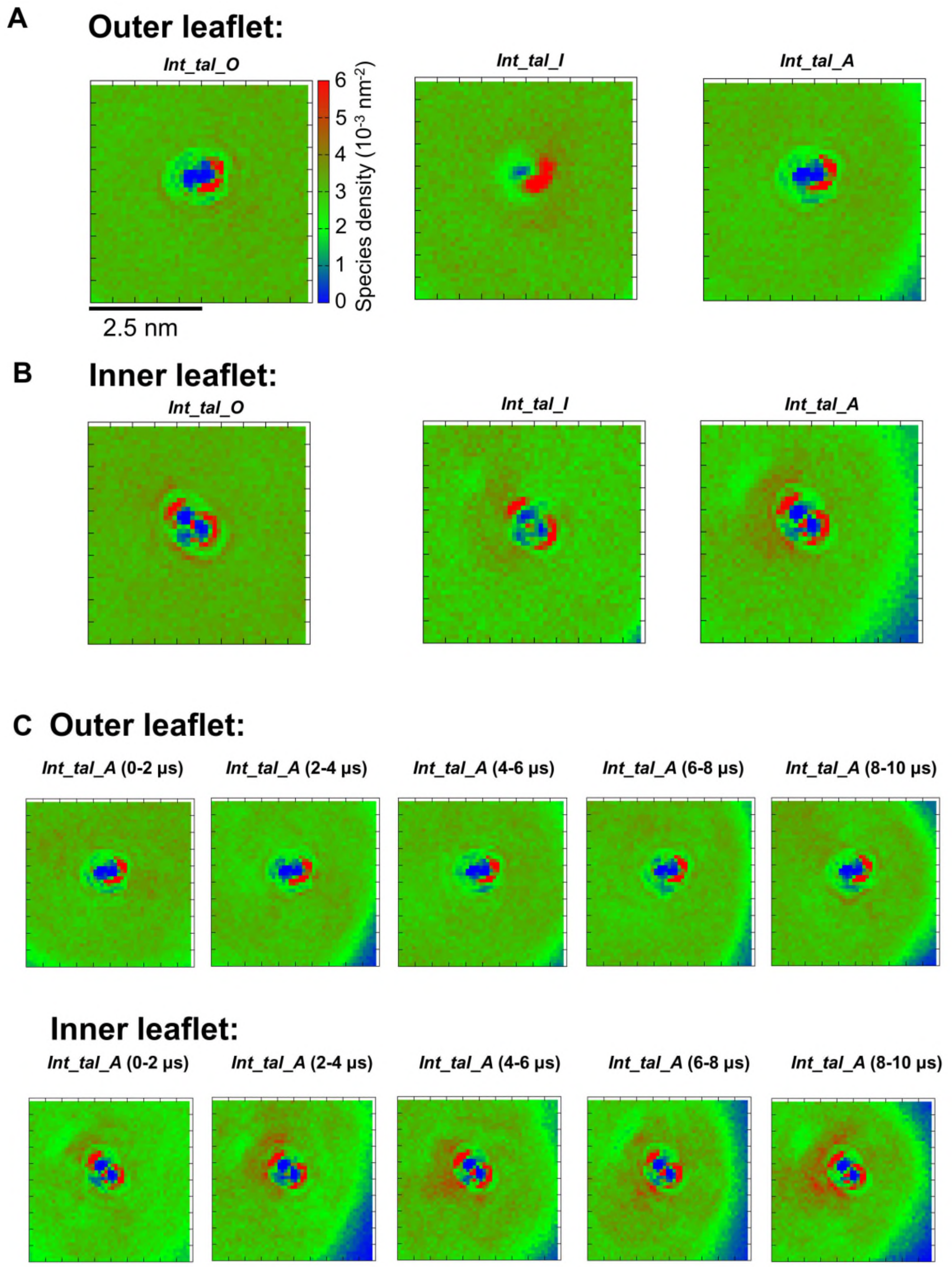


*Supplementary Fig. S5:*
A,B Two-dimensional average cholesterol densities (of the ROH particles) around the integrin/F2-F3 complex for the outer (A) and inner (B) leaflets of the *3* different simulation systems. C. The same densities are shown for the 5 sub-trajectories (5 x 2 µs) of the *int_tal_A* simulation. The diagrams show the probability of finding cholesterol at a given point in the bilayer plane around the protein. Blue represents low probability through red for a high probability.

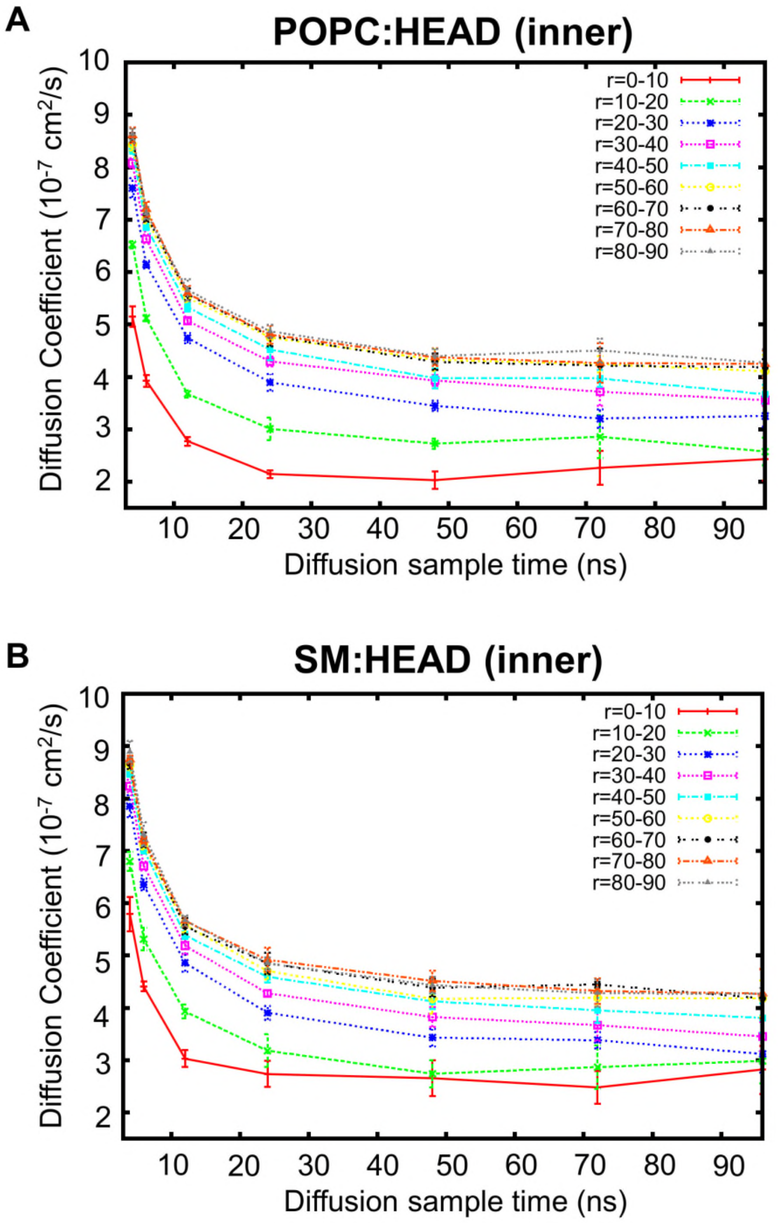


*Supplementary Fig. S6:*

A,B Diffusion coefficient of the POPC and SM headgroups in the inner leaflet of the *int_tal_O* simulation as a function of observation time within a 1 nm radius around the protein. The errors were calculated as the standard deviations from 5 sub-trajectories (5 x 2 μs).

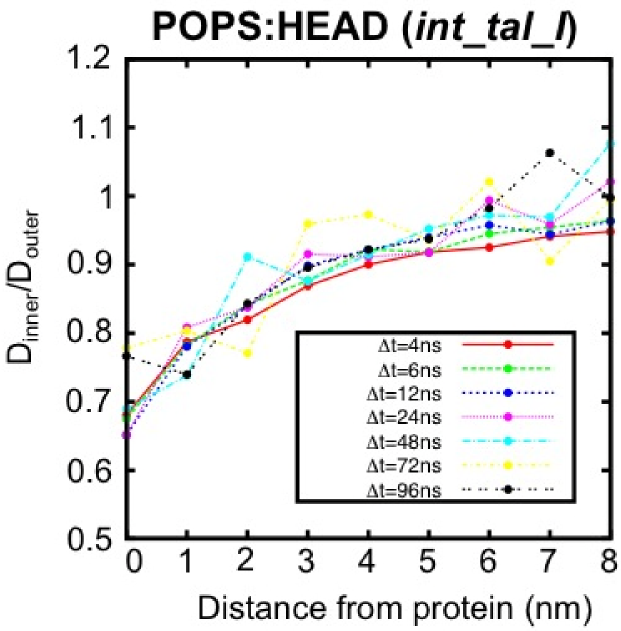


*Supplementary Fig. S7:*
Diffusion coefficient ratio of the POPS headgroups for the inner and the outer leaflets in the *int_tal_I* simulation as a function of distance from the protein over different timescales (Δt).

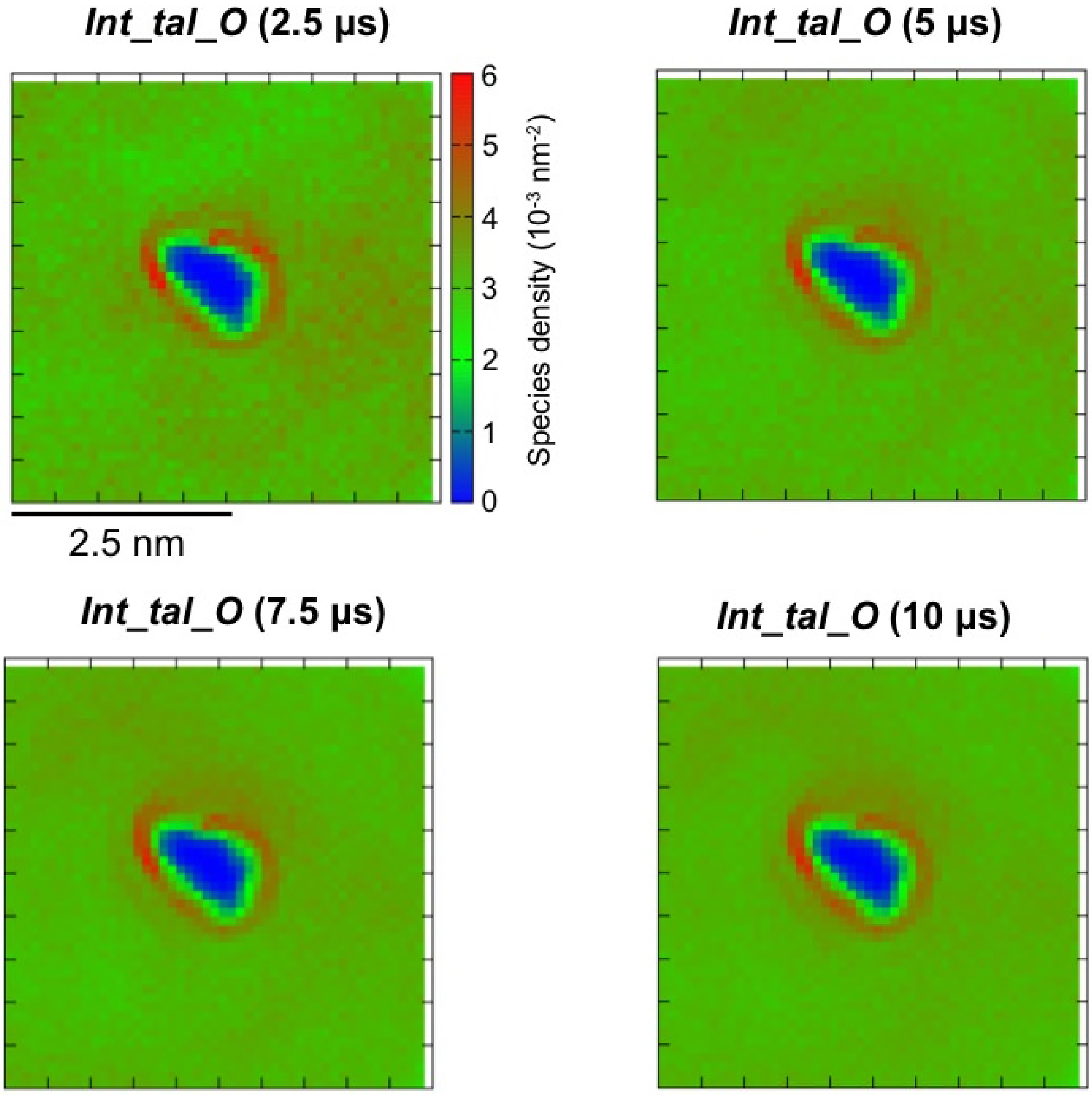


*Supplementary Fig. S8:*
Convergence analysis. Two-dimensional averages of lipid headgroup densities of POPC lipids in the *tal_αβ_O* simulation are shown at 2.5 µs, 5 µs, 7.5 µs and 10 µs of the simulation.
